# The lipid biology of sepsis

**DOI:** 10.1016/j.jlr.2021.100090

**Published:** 2021-06-01

**Authors:** Kaushalya Amunugama, Daniel P. Pike, David A. Ford

**Affiliations:** 1 Edward A. Doisy Department of Biochemistry and Molecular Biology, Saint Louis University School of Medicine, St. Louis, MO, USA; 2 Center for Cardiovascular Research, Saint Louis University School of Medicine, St. Louis, MO, USA

**Keywords:** sepsis pathogenesis, lipid metabolism, biomarkers, inflammation, resolution, energy imbalance, bioactive lipids, fatty acids, cholesterol, prognostics, AA, arachidonic acid, AEA, N-arachidonoylethanolamine, 2-AG, 2-Arachidonoylglycerol, ARDS, acute respiratory distress syndrome, ATX, autotaxin, CEPT, cholesteryl ester transfer, Cer, ceramide, 2-ClFA, 2-chlorofatty acid, 2-ClFALD, 2-chlorofatty aldehyde, CLP, cecal ligation puncture, 2-ClPA, 2- chloropalmitic acid, 2-ClSA, 2-chlorostearic acid, COX, cyclooxygenase, cPLA_2_, cytosolic PLA_2_, CS, cecal slurry, CYP450, cytochrome P450, cys-LTs, cysteinyl leukotrienes, DHET, dihydroxyeicosatrienoic acid, DPA, docosapentaenoic acid, EET, epoxy-eicosatrienoic acid, HOCl, hypochlorous acid, LOX, lipoxygenase, LPA, lysophosphatidic acid, LPC, lysophosphatidylcholine, LPCAT, lysophosphatidylcholine acyltransferase, LPS, lipopolysaccharide, LTA, lipotechoic acid, LTB_4_, leukotriene B_4_, MPO, myeloperoxidase, PAF, platelet-activating factor, PC, phosphatidylcholine, PCSK9, proprotein convertase subtilisin/kexin type 9, PLA_2_, phospholipase A_2_, rHDL, recombinant HDL, Rv, resolvin, RvD1-RvD6, D series resolvins, RvE1-RvE3, E series resolvins, RvT1-RvT4, T series resolvins, 10S,17S- diHDHA, 10(S),17(S) dihydroxy docosahexaenoic acid, sEH, soluble epoxide hydrolase, SIRS, systemic inflammatory response syndrome, S1P, sphingosine-1-phosphate, sPLA_2_, secretory phospholipase A_2_, Sphk, sphingosine kinase, SPMs, specialized pro-resolving mediators, SOFA, sequential organ failure assessment, TC, total cholesterol, TG, triglycerides, TLR, Toll-like receptors, TPPU, N-[1-(1-oxopropyl)-4-piperidinyl]-N′-[4-(trifluoromethoxy)phenyl]-urea, TXB2, thromboxane B2

## Abstract

Sepsis, defined as the dysregulated immune response to an infection leading to organ dysfunction, is one of the leading causes of mortality around the globe. Despite the significant progress in delineating the underlying mechanisms of sepsis pathogenesis, there are currently no effective treatments or specific diagnostic biomarkers in the clinical setting. The perturbation of cell signaling mechanisms, inadequate inflammation resolution, and energy imbalance, all of which are altered during sepsis, are also known to lead to defective lipid metabolism. The use of lipids as biomarkers with high specificity and sensitivity may aid in early diagnosis and guide clinical decision making. In addition, identifying the link between specific lipid signatures and their role in sepsis pathology may lead to novel therapeutics. In this review, we discuss the recent evidence on dysregulated lipid metabolism both in experimental and human sepsis focused on bioactive lipids, fatty acids, and cholesterol as well as the enzymes regulating their levels during sepsis. We highlight not only their potential roles in sepsis pathogenesis but also the possibility of using these respective lipid compounds as diagnostic and prognostic biomarkers of sepsis.

## Graphical abstract

**Figure:**
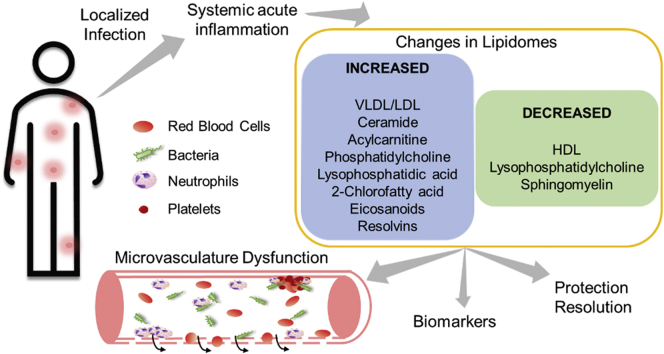

Sepsis is a systemic inflammatory disease with high morbidity and mortality caused by a dysregulated host immune response to an infection. Sepsis strikes more than 1.5 million patients in the United States per year, resulting in 250,000 deaths annually. Globally, sepsis has been estimated to account for 20% of all deaths (1). In the United States, the most common sources of sepsis are bacterial pneumonia, urinary tract infections, and abdominal infections (2). The dysregulated and systemic immune response during sepsis can lead to host tissue injury, organ failure, and death. Central to sepsis-elicited organ injury and mortality are complex interactions between blood cells, microbes, and endothelium, resulting in multiorgan microcirculatory failure. In sepsis, activated endothelial cells lead to an increased propensity for leukocyte and platelet adhesion and increased permeability barrier dysfunction (3, 4). In addition, the coagulation cascade is systemically activated, and endogenous anticoagulant factors are unable to sufficiently match the increased demand to appropriately control clotting (5). Platelets contribute to microthrombi formation, in addition to propagating proinflammatory signaling (6). Collectively, these changes to the endothelium and microvascular environment in sepsis lead to decreased oxygenated blood flow to target organs, contributing to organ failure. In addition to hypoxic injury, tissues can be damaged directly by the release of toxic leukocyte-derived proinflammatory mediators (7, 8). Dysfunctional neutrophils in sepsis are hyperinflammatory (9), have significant defects in localization and trafficking (10), and are resistant to regulatory apoptotic signaling pathways (11). These dysfunctional alterations further drive tissue damage and organ injury through the release of reactive oxygen species and proteases. Treatments for sepsis are limited to antibiotics targeting the infectious source and supportive care for organ dysfunction. Delineating the mechanisms underlying sepsis-induced microcirculatory collapse and the dysregulated immune response may lead to both improved therapeutic interventions and new biomarkers indicative of early sepsis and organ-specific risk.

Alterations in lipid metabolism and the activation of lipid signaling pathways are components of the complex milieu underlying the pathophysiological sequelae of sepsis. Lipid mediators play an important role in the proinflammatory and counterregulatory anti-inflammatory changes in the microvasculature in sepsis. Some of these lipids display significant inherent bioactivity, whereas others may be by-products and reflect important metabolic alterations in a septic patient. A better understanding of the various lipid species, their production and metabolism, and their bioactivity has the potential to lead to the development of early biomarkers and therapeutic targets in sepsis. This review focuses on the role of lipid alterations during sepsis and the potential of lipids to provide both new biomarkers and therapeutic targets to improve outcomes in sepsis.

## Eicosanoids and specialized proresolving mediators

**Fig. 1:**
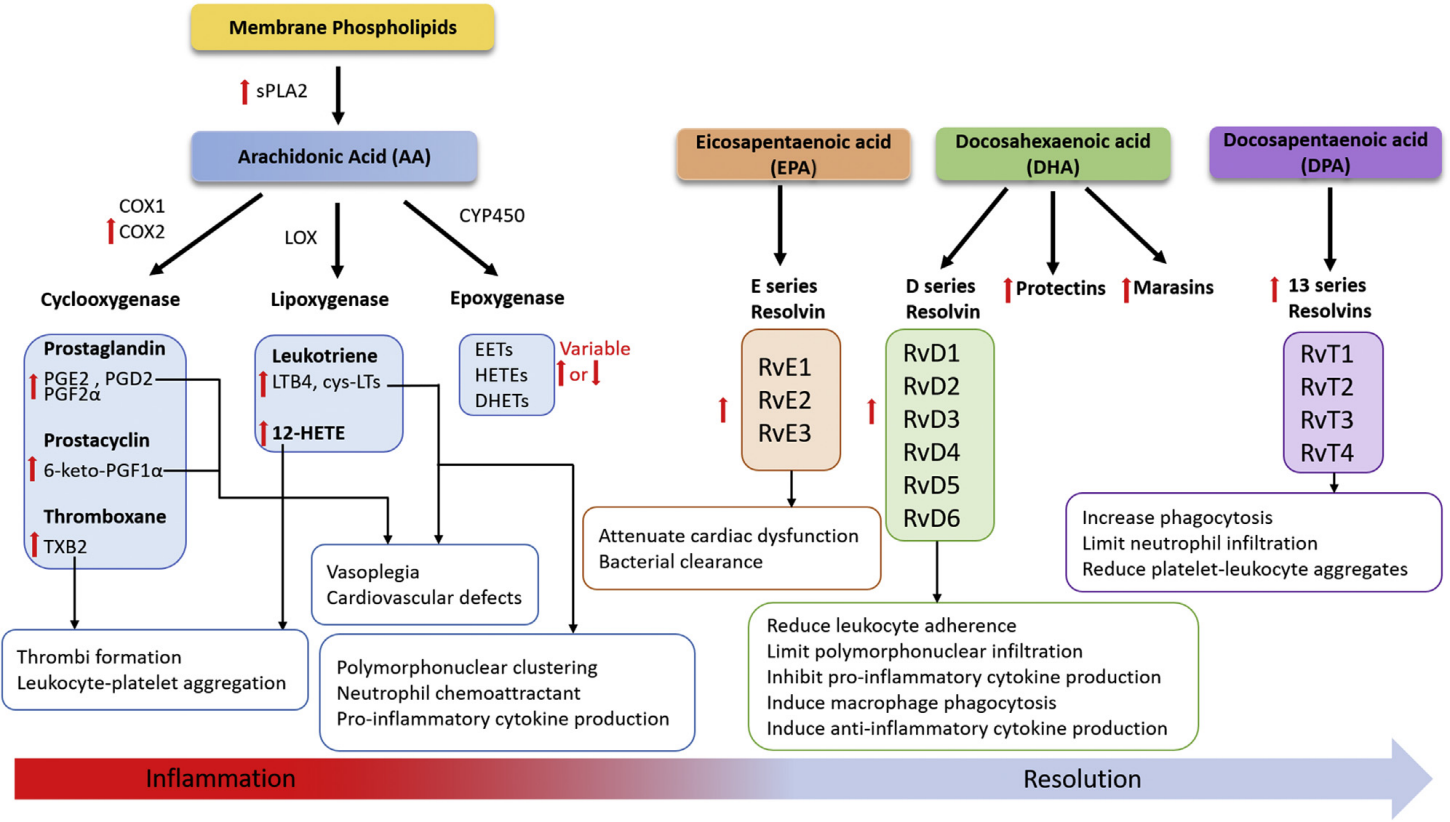
Eicosanoids and SPMs in sepsis. Sepsis leads to either increased or decreased (red arrows) eicosanoid and SPM production. Secretory phospholipase A2 (sPLA_2_) activity is increased to liberate arachidonic (AA) from plasma phospholipids, and then inducible cyclooxygenase 2 (COX2) activity results in elevated production of cyclooxygenase-derived eicosanoids. Eicosanoids are mediators of proinflammatory mechanisms during sepsis. Proinflammatory mechanisms are counter-regulated by SPMs, which aid in the recovery of sepsis. EPA, DHA, and DPA derived SPMs are increased leading to reduction in inflammatory damage. COX1/2, cyclooxygenase 1/2; CYP450, cytochrome P450; cys-LT, cysteinyl leukotriene; DHET, dihydroxyeicosatrienoic acid; EET, epoxyeicosatrienoic acid; LOX, lipoxygenase; LTB_4_, leukotriene B_4_; PGD_2_, prostaglandin D_2_; PGE_2_, prostaglandin E_2_; PGF_2α_, prostaglandin F_2α_; RvD1-6, resolvin D 1–6; RvE1-3, resolvin E 1–3; RvT 1–4, resolvin T 1–4; sPLA_2_, secretory phospholipase A_2_; TXB_2_, thromboxane B2.

Leukocytes are recruited to sites of infection and inflammation, contributing to a multifactorial proinflammatory response, a component of which is the release of proinflammatory cytokines and eicosanoid mediators. This initial proinflammatory cascade activates antimicrobial systems intended to kill pathogens (12, 13). However, this response requires self-limitation to prevent exaggerated immune reactions from damaging host tissue. Thus, the proinflammatory response is balanced against an anti-inflammatory response mediated by anti-inflammatory cytokines and a class of lipids known as specialized proresolving mediators (SPMs). The proinflammatory eicosanoids are a family of bioactive lipids derived from arachidonic acid (AA). AA is released from membrane phospholipids by the phospholipase A_2_ (PLA_2_) enzymes (14, 15, 16). Liberated AA is then oxidized into eicosanoids by a variety of enzymes, including the cyclooxygenase (COX), lipoxygenase (LOX), and cytochrome P450 (CYP450) families. In an analogous manner, omega-3 fatty acids, including DHA, docosapentaenoic acid (n-3DPA), and EPA, exist in esterified pools in membrane phospholipids and are also released by PLA_2_. These liberated omega-3-fatty acids are themselves subsequently metabolized to yield the SPMs (Fig. 1).

The roles of eicosanoids and SPMs have been investigated in sepsis and inflammatory processes (17, 18, 19, 20, 21). Mice subjected to the cecal ligation puncture (CLP) model of sepsis accumulate thromboxane B2 (derived from biologically active thromboxane A2) and 12-HETE, major platelet-derived eicosanoids that promote thrombus formation by inducing platelet activation, platelet aggregation, and vasoconstriction (22). The accumulation of these prothrombotic lipids in CLP sepsis is subsequently accompanied by increased thrombi formation in lung capillaries and enhanced monocyte, neutrophil, and platelet aggregates. Thrombosis and thrombocytopenia are common complications in human patients with sepsis owing to overconsumption of platelets for aggregation or adhesion to the endothelium and leukocytes (23, 24). Other studies also demonstrated similar elevations in eicosanoid lipid mediators during sepsis across species. Serum levels of the prostaglandins PGE_2_ and PGD_2_ are almost doubled in human subjects with sepsis. This elevated AA-derived eicosanoid production in human sepsis is accompanied by increased secretory PLA_2_ group IIA and COX-2 activity (25). Similarly, both CLP-septic and lipopolysaccharide (LPS)-treated mice have increased plasma PGE_2_ and PGE_2_ levels (26). Prostacyclin levels are also increased in sepsis, indicated by increased stable metabolite levels of 6-keto-PGF_1α_ in plasma and perivascular adipose tissues of septic rats (27, 28). These studies also suggested that prostacyclin has a role in sepsis-induced vasoplegia and cardiovascular failure.

LPS administration and CLP sepsis in mice results in variable responses in plasma levels of various hydroxy-FA, dihydroxy-FA, and epoxy-FA eicosanoid species (26). The dihydroxy-FA species 14,15-DiHETE and 14,15-DiHETrE are elevated in plasma, suggesting targeting of EPA and AA acid, respectively, by CYP450 and then epoxygenase. Epoxy-eicosatrienoic acids (EETs) are metabolized to dihydroxyeicosatrienoic acids (DHETs) by soluble epoxide hydrolase (sEH) (29), and targeting this pathway may be important in sepsis owing to the anti-inflammatory properties of EETs. In CLP mice, sEH protein expression is increased in the brain tissues, mainly in endothelial cells, and is associated with cognitive deficits of sepsis-associated encephalopathy. Pharmacological inhibition of sEH with N-[1-(1-oxopropyl)-4-piperidinyl]-N′-[4-(trifluoromethoxy)phenyl]-urea (TPPU) in septic mice has improved the blood-brain barrier function and cognitive functions (30). TPPU has been shown to be a potent inhibitor of sEH resulting in increased EET levels and reduced inflammation (31). Recently, TPPU treatment of CLP mice was shown to increase EET levels, reduce multiorgan dysfunction, and reduce inflammation (32). The use of TPPU or other sEH inhibitors has yet to go forward in clinical trials, but this approach appears to be a promising potential treatment to be further explored.

Leukotriene B_4_ (LTB_4_) is a potent neutrophil chemoattractant and enhances phagocytosis (33). In mice subjected to fungal sepsis, complement activation induces LTB_4_-elicited intravascular neutrophil clustering (34). Both elevated LTB_4_ and cysteinyl leukotrienes are associated with pulmonary hemorrhage, hypoxemia, neutrophil infiltration, lung damage, and poor clinical outcomes in septic mice (35). Genetic ablations targeting either the LTB_4_ receptor or 5-lipoxygenase enzyme have been used to reduce leukotriene biological activity in sepsis models (34, 35). These manipulations attenuate inflammation and neutrophil recruitment in the lungs and the associated lung injury in septic mice. Furthermore, pharmacological inhibition of the LTB_4_ receptor increases anti-inflammatory cytokine IL-10 production in lungs and plasma of septic mice (35).

Significant alterations in SPM metabolism are evident in sepsis, suggesting defects in the resolution phase of acute inflammation. Resolvins (Rvs) are a class of SPMs and can be categorized into several subclasses: D series resolvins (RvD1-RvD6) derived from DHA, E series resolvins (RvE1-RvE3) derived from EPA, and T series resolvins (RvT1-RvT4) derived from n-3 DPA (Fig. 1). Resolvin concentrations are dramatically altered in human sepsis nonsurvivors and may potentially have prognostic value (21). Plasma RvD1-RvD6 and Maresin-1 are elevated in patients with sepsis, and significantly elevated concentrations of RvD5 are associated with sepsis nonsurvival (19, 36). Plasma RvT1-RvT4 and RvE1-RvE3 are also significantly elevated in patients with sepsis (36). Riché *et al.* (37) investigated temporal changes in resolvins in patients with sepsis. At the onset of sepsis, plasma RvD1 and RvD5 are at lower concentrations and are accompanied by an upregulated proinflammatory state. Following intensive care treatment and during long-term recovery, resolvin levels are elevated, potentially reflecting an attempt to counterbalance ongoing inflammation (37). Moreover, upregulated plasma protectin D1 isomer, 10(S),17(S) dihydroxy docosahexaenoic acid (10S,17S-diHDHA) levels correlate with acute respiratory distress syndrome (ARDS) development in patients with sepsis (19). In addition to a potential prognostic/diagnostic role, resolvins may also, in part, mediate sepsis pathophysiology, as RvD2 supplementation in CLP septic mice enhances clearance of bacteria, prevents sepsis-induced lethality, reduces neutrophil infiltration into the peritoneum, and inhibits proinflammatory cytokine production (38, 39). Intraperitoneal administration of RvT in *Escherichia coli*-infected mice protects mice from hypothermia, decreases neutrophil recruitment to sites of inflammation, and increases bacterial phagocytosis (36).

Taken together, it is well established that oxidation products derived from n-3 and n-6 fatty acids are produced in human and animal models of sepsis. These oxidation products have both proinflammatory and anti-inflammatory roles, and they have major roles in the complex biochemistry that drives sepsis pathophysiology. The complex nature of inflammatory balance during sepsis, in which the early-onset proinflammatory condition is followed by a compensatory immunosuppressive response, makes the role of these oxidized lipids crucial targets of further investigation, since they likely have a key mechanistic role in this balance. These anti- and proinflammatory roles as they relate to sepsis inflammation and resolution are summarized in Fig. 1.

## Lysophosphatidylcholine

**Fig. 2:**
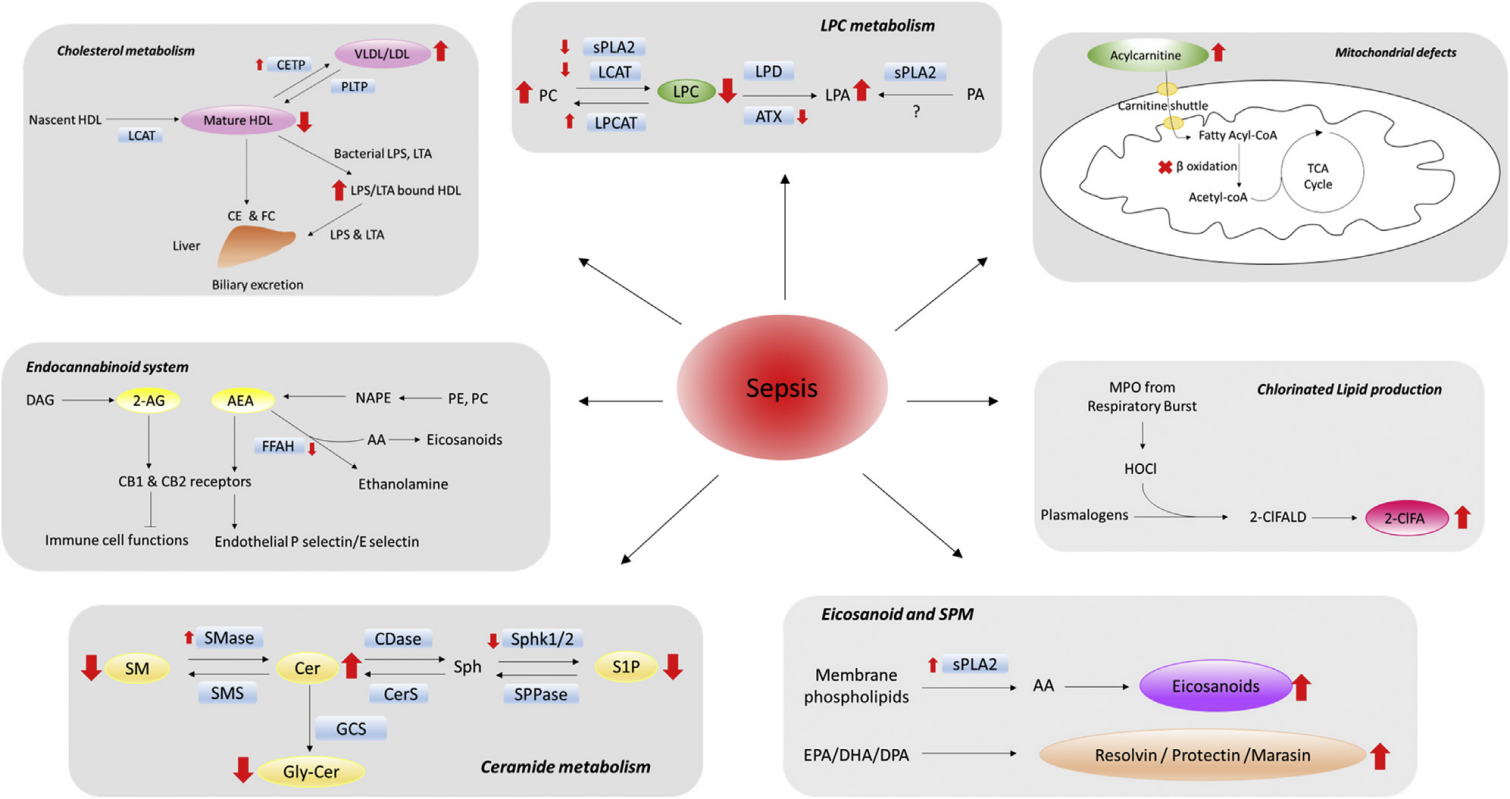
Lipids altered in sepsis. Sepsis induces alterations in lipid signaling mechanisms (gray boxes). Red arrows indicated up- or downregulated lipids (big red arrows) and enzymes (small red arrows) relevant to respective lipid biosynthesis that are evident in sepsis. 2-AG, 2-arachidonoylglycerol; 2-ClFA, 2-chlorofatty acid; 2-ClFALD, 2-chlorofatty aldehyde; AA, arachidonic acid; AEA, N-arachidonoylethanolamine; ATX, autotaxin; CB1/2, cannabinoid receptor 1/2; CDase, ceramidase; CE, cholesterol ester; Cer, ceramide; CerS, ceramide synthase; CETP, cholesteryl ester transfer protein; DAG, diacylglycerol; FC, free cholesterol; FFAH, fatty acid amide hydrolase; GCS, glucosylceramide synthase; Gly-Cer, glycosylated ceramide; HOCl, hypochlorous acid; LPA, lysophosphatidic acid; LPC, lysophosphatidylcholine; LPCAT, lysophosphatidylcholine acyltransferase; LPD, lysophospholipase D; LPS, lipopolysaccharide; LTA, lipoteichoic acid; MPO, myeloperoxidase; NAPE, N-acyl phosphatidylethanolamine; PA, phosphatidic acid; PC, phosphatidylcholine; PE, phosphatidylethanolamine; PLTP, phospholipid transfer protein; S1P, sphingosine-1-phosphate; SMase, sphingomyelinase; SMS, sphingomyelin synthase; Sph, sphingosine; Sphk1/2, sphingosine kinase 1/2; sPLA_2_, secretory phospholipases A2; SPPase, S1P-phosphatase.

Products of PLA_2_ activity are fatty acids and lysophospholipids. Phosphatidylcholine (PC) hydrolysis by PLA_2_ yields fatty acid and lysophosphatidylcholine (LPC). LPC levels are tightly regulated by PLA_2_ activity and several isozymes of lysophosphatidylcholine acyltransferase (LPCAT), which are responsible for the reacylation of LPC (40). Alternatively, LPC can be further hydrolyzed by phospholipase D or autotaxin (ATX) to produce lysophosphatidic acid (LPA) (Fig. 2). LPC contributes to inflammation by increasing chemokine production and activating endothelium, neutrophils, monocytes, macrophages, and lymphocytes. Total serum LPC levels are significantly lower in human sepsis (21, 41), and other studies demonstrated that the major LPC molecular species (16:0 LPC, 18:0 LPC, 18:1 LPC, and 18:2 LPC) are decreased 50% in serum and plasma of patients with sepsis (42, 43). Less common species including 15:0 LPC, 18:3 LPC, 20:3 LPC, 20:4 LPC, and 20:5 LPC are also decreased in plasma and erythrocytes isolated from patients with sepsis (44).

Serum LPC concentrations may have utility in predicting sepsis severity. In human patients with sepsis, serum and plasma LPC concentrations increase over time in survivors but not in nonsurvivors, while persistently lower plasma LPC levels associate with 28- and 90-day mortality (45, 46). Of interest, lower plasma 24:0 LPC at day 7 is a strong predictor of 90-day mortality in patients with sepsis (45, 46). In addition, the molar ratio of LPC/PC is markedly decreased both in plasma and erythrocytes of patients with sepsis compared with heathy subjects (42, 44). Moreover, plasma LPC/PC is significantly decreased in sepsis nonsurvivors compared with survivors, and this ratio can predict 30-day mortality (42). Serum LPC may predict the source of infection in sepsis, potentially guiding appropriate antimicrobial therapy. For instance, elevated 26:1 LPC discriminates patients sick due to community-acquired pneumonia from those sick from other sources. In addition, LPC is lower in bacteremic compared with nonbacteremic sepsis (41, 47).

Ahn *et al.* (48) investigated mechanisms responsible for this decrease in plasma LPC levels in sepsis by assessing precursors, metabolites, and relevant enzymes in mice subjected to CLP. LPC was the only examined lipid that was decreased, whereas PC and LPA levels were increased in CLP mice. The activities of enzymes responsible for LPC production in plasma, lecithin-cholesterol acyltransferase and sPLA_2_, were greatly attenuated in these mice. Upregulated plasma LPCAT1-3 activity, accompanied with higher PC concentration, suggests that sepsis triggers LPC conversion to PC. Of interest, plasma ATX levels were decreased, yet plasma LPA levels were increased in septic mice, suggesting that mechanisms responsible for the reacylation of LPA were not activated, unlike those for LPC reacylation (48).

Exogenous LPC enhances neutrophil bacterial killing and augments monocyte chemotaxis in vitro (49, 50). Exogenous LPC acts through the G protein–coupled receptor, G2A, to inhibit the production of the proinflammatory cytokines IL-1β and TNF-α (51). In vivo, exogenous LPC increases bacterial clearance, reduces organ damage, and improves survival rates in septic mice and rats when administered alone or in combination with an antimicrobial agent (51, 52, 53, 54).

The role of LPC in sepsis needs to be further explored, as plasma and serum levels may provide improved prognostic biomarkers in human sepsis. Although animal studies suggest that increasing plasma LPC with exogenous administration is protective in sepsis, this would need to be carefully examined further in animal models to determine critical levels that are therapeutically advantageous. Alternately, pathways downstream of LPC could be identified to replicate the potentially beneficial role of LPC in sepsis, as LPC also has many deleterious consequences, including proinflammatory, atherogenic, and arrhythmogenic effects (55, 56, 57).

## Phospholipase A_2_

Both secretory phospholipase A_2_ (sPLA_2_) and cytosolic PLA_2_ (cPLA_2_) have roles in regulating arachidonic acid release and subsequent oxylipid production as well as lysophospholipid production and degradation. Several studies have shown an upregulation of sPLA_2_ and cPLA_2_ in serum and plasma of patients with sepsis (58, 59, 60). cPLA_2_ activity in peripheral neutrophils isolated from patients with sepsis is increased 2-fold compared with activity in control neutrophils (61). This increased neutrophil cPLA_2_ activity returns to normal levels as sepsis resolves (4). Using a genetic knockout approach in mice, cPLA_2-_α knockout mice subjected to CLP have been shown to have an attenuated inflammatory response to sepsis. These mice had lower PGE_2_, LTB_4_, and 6-keto-PGF_1α_ levels in peritoneal lavage fluid compared with control CLP mice. However, genetic ablation of cPLA_2-_α did not increase survival rates in these sepsis studies (62). It should also be appreciated that cPLA_2-_α is known to have lysophospholipase activity, which can modulate LPC levels and may contribute to deleterious effects in sepsis (63).

Of the two groups of sPLA_2_, subtype A (of the ten subtypes) of class II (i.e., sPLA_2_-IIA) has been shown to be associated with sepsis (64). Serum sPLA_2_-IIA activity has a positive correlation with early sepsis diagnosis in adult patients as well as with bacterial etiology (64, 65). Similarly, in neonatal sepsis plasma, sPLA_2_ activity was elevated, whereas infants with respiratory distress syndrome had higher sPLA_2_ activity (66). Furthermore, sPLA_2_-IIA has been shown to promote antibacterial activity (67, 68, 69). In other studies, reducing sPLA_2_ and cPLA_2_ activity with oligonucleotides against sPLA_2_-IIA and cPLA_2-_α led to increased survival in septic rats (70). Although sPLA_2_-IIA inhibitors have some beneficial effects in animal models of sepsis, clinical trials of the sPLA_2_-IIA inhibitor, LY315920NA/S-5920, failed to improve outcomes in human sepsis (71).

## Platelet-activating factor

Platelet-activating factor (PAF) also likely contributes to sepsis pathophysiology. PAF is synthesized either by a remodeling pathway or by de novo biosynthesis (72, 73, 74, 75, 76). Proinflammatory signals such as LPS and thrombin induce PAF production by neutrophils and endothelial cells (77, 78, 79). Many cells and tissues express the PAF receptor, including monocytes, neutrophils, B-cells, platelets, endothelial cells, and lung tissue (80, 81). PAF can bind to, activate, and induce the aggregation of leukocytes and platelets (82, 83, 84) as well as reactive oxygen species production, NETosis, and platelet adherence (85, 86, 87, 88, 89). Considering its effects on cells in the microvasculature environment, it is unsurprising that PAF and enzymes responsible for PAF metabolism are implicated in sepsis (90).

In endotoxemia models, PAF levels associate with poor clinical outcomes. Overexpression of the PAF receptor increases LPS-induced mortality, as does PAF administration directly after LPS injections (91, 92). Conversely, enhancement of PAF metabolism leads to improved outcomes. PAF is metabolized by PAF-acetylhydrolase, which is expressed by many tissues and also associates with plasma lipoproteins (93, 94, 95, 96). Recombinant PAF-acetylhydrolase improves bacterial clearance in CLP models and survival after both CLP and endotoxemia (97, 98). In addition, PAF receptor blockade improves survival, leukocyte migration, and pathogen clearance after CLP (99).

Data from human patients with sepsis reinforces the association between PAF and mortality. Patients with sepsis have increased platelet-associated PAF compared with other patients (100). One study demonstrated that PAF in sepsis nonsurvivors has an increased half-life in plasma compared with sepsis survivors or healthy subjects. The same study also demonstrated that patients with sepsis have reduced plasma PAF-acetylhydrolase activity, increased plasma PLA_2_ activity, and decreased plasma lyso-PAF concentrations (101). Decreased PAF-acetylhydrolase activity is associated with multiorgan failure (102). Conversely, increased activity of PAF-acetylhydrolase 7 days following the diagnosis of ARDS is associated with survival (103). Unfortunately, however, recombinant PAF-acetylhydrolase did not improve sepsis outcomes in a phase III clinical trial (104).

## Sphingolipids

Sphingolipids have profound functions in cell signaling and regulating inflammatory responses (105). Cellular processes regulated by sphingolipids include lymphocyte trafficking, mediating pro- and anti-inflammatory effects, and maintaining endothelial barrier integrity (105, 106). Investigations focusing on the role of sphingolipids in sepsis have predominantly focused on SM, ceramide (Cer) and sphingosine-1-phosphate (S1P) as biomarkers of sepsis outcomes. Several studies have shown decreased plasma levels of SM in patients with sepsis and decreased plasma levels of SM in patients with sepsis with acute lung injury (44, 107). A statistical model using combined serum 22:3 SM and 24:0 LPC concentrations discriminates sepsis from systemic inflammatory response syndrome (SIRS), a nonspecific state of systemic inflammation that does not require an infectious source (47). Other studies demonstrate that nonsurvivors within 28 days of sepsis diagnosis had a marked decline in plasma 20:2 SM compared with survivors (46). However, CLP rodents do not have decreased levels of plasma SM. Rather, 24 h after CLP, rodents have increased SM levels (108, 109). This is just one of many examples of the differences between human and rodent sepsis, which have made dissection of biochemical mechanisms in rodents difficult to translate into humans and have generated controversy regarding the applicability of rodent models of sepsis (110, 111).

Others have assessed the sphingomyelinase product, Cer, in sepsis. Both total Cer content and individual molecular species of Cer, including 16:0, 18:0, 20:0, 22:1, and 24:1, are elevated in plasma from patients with sepsis (42, 112). Furthermore, increased levels of Cer in peripheral blood mononuclear cells isolated from patients with sepsis are associated with the incidence of multiple organ dysfunction (113). The product/precursor ratio of Cer/SM has also been shown to progressively increase from day 1 to day 11 in human sepsis nonsurvivors (42).

S1P is a lipid mediator that regulates many physiological and pathological processes (114). Serum or plasma S1P levels in patients with septic are approximately 50% lower compared with healthy controls (115, 116, 117, 118), and S1P concentrations inversely correlate with Cer concentration and sequential organ failure assessment (SOFA) score, an assessment of the severity of critically ill patients (112). In blood, S1P is bound to apo-M on HDL particles and albumin (119). A marked loss of HDL and apo-M is observed in both septic patients and animal models of sepsis, which may account for decreased plasma S1P levels (116, 117). Diminished S1P levels likely have a role in lung injury during sepsis as treatments with S1P as well as its analog, FTY720, have been shown to improve barrier function (120, 121).

Plasma acid sphingomyelinase and neutral sphingomyelinase activity in platelets are increased in septic patients, suggesting an increase in SM metabolism in sepsis (112, 122). Treating endothelial cells with serum from septic patients resulted in endothelial cell SM loss accompanied by a sharp and rapid production of Cer, which then is further metabolized to glycosylated Cers (122). However, these results were not observed by Goeritzer *et al.* (123), who observed decreases in brain endothelial cell levels of Cer and an associated loss of barrier function after treatment with septic patient serum. Decreased S1P levels in human sepsis is likely the result of reduced sphingosine kinase (Sphk1 and Sphk2) activity (112). Sepsis-induced cardiac dysfunction, lung edema, and declined anti-inflammatory responses can be reversed by genetic ablation or pharmacological inhibition of sphingomyelin phosphodiesterase (SMPD1) and Sphk in septic rodents (118, 124, 125, 126). Overall, these investigations indicate that dysregulated sphingomyelin and Cer homeostasis have a significant role in sepsis pathogenesis.

## Plasma lipoproteins, cholesterol, and triglycerides

HDL can bind to LPS and modulate infection and inflammation. LPS has greater affinity for HDL than it has for VLDL and LDL (127). It has been suggested that phosphates and the diglucosamine backbone, the functional groups of LPS, interact with HDL particles (128). Henning *et al.* (129) demonstrated that LPS binds to apo-A1, the major protein found in HDL particles. HDL can also bind to lipoteichoic acid (LTA), a component of gram-positive bacterial cell walls (130). The binding of HDL to either LPS or LTA interferes with its ability to bind to Toll-like receptors (TLRs) in macrophages (131). HDL also impairs TLR signaling by interfering with lipid rafts and by inducing ATF3, a key transcriptional modulator of innate immune response (131, 132). ATF3 activation by HDL acts as a negative-feedback loop to downregulate TLR-driven inflammatory responses (131). In addition to its endotoxin detoxification function, HDL modulates immune cell responses. HDL inhibits integrin CD11b and cytokines in monocyte and neutrophils (133, 134). It also suppresses neutrophil adhesion, spreading, and migration (134). HDL protects endothelium by modulating eNOS-dependent vascular tone, decreasing leukocyte adhesion to the endothelium, and promoting COX2 and prostacyclin to inhibit thrombosis (135, 136, 137). Nofer *et al.* demonstrated that HDL binds to S1P and interacts with S1P receptors on endothelium to activate eNOS in an Akt-dependent manner, whereas Yuhanna *et al.* demonstrated that HDL binds to scavenger receptor B1 to stimulate eNOS activity, resulting in vasorelaxation (135, 138). A recent study has shown that HDL-S1P levels are negatively correlated with endothelial dysfunction in septic patients and animal models (139). HDL-S1P administration also reduces LPS-induced acute lung injury in rats (139). Collectively, the protective actions of HDL on endothelium and sequestration of endotoxin suggest that HDL is protective during sepsis.

However, plasma HDL levels are decreased in human sepsis. An approximate 30% drop of HDL occurs in patients with sepsis on the day of hospital admission (usually defined as day 0) compared with healthy controls (140, 141). Decreased HDL levels are associated with increased sepsis mortality and can predict multiorgan dysfunction (142, 143, 144, 145). Furthermore, longitudinal studies reveal that serum HDL continually declines in sepsis nonsurvivors at day 0, day 3, and day 10 compared with survivors (144). Septic patients who have HDL concentrations lower than 25.1 mg/dl on hospital admission are highly susceptible to adverse outcomes, such as requirement of intensive care unit care, development of multiple- or single-organ dysfunctions (respiratory, circulatory, hepatic, or renal), and mortality (143). Low HDL has been associated with the development of sepsis-associated acute kidney injury and long-term declines in estimated glomerular filtration rate, suggesting that HDL may serve as biomarker of renal dysfunction in sepsis (146). HDL levels in septic patients are inversely correlated with proinflammatory cytokines such as TNF-α and IL-6 (144, 147). This decrease in HDL levels appears to be specific for systemic inflammation due to infection, as no decreases are observed in trauma, local infection, or SIRS (148, 149). Even after clinical recovery from sepsis, HDL remains at lower concentrations with marked reduction of anti-inflammatory properties, leaving the patients more vulnerable to secondary infection (141).

Small HDL particles have anti-inflammatory and antioxidant properties, and HDL particle size is altered in sepsis. Following low-dose endotoxemia in humans, the number of small and medium-sized HDL particles are decreased (150, 151). Patients with sepsis have shifts to large HDL particle sizes from intermediate and small HDL particles (140). This shift is due to impaired HDL maturation and increased catabolism of the small and medium-sized HDL particles. During endotoxemia, HDL particle remodeling is associated with increased endothelial lipases and sPLA_2_ and decreased plasma cholesteryl ester transfer protein (CETP) and lecithin-cholesterol acyltransferase activity (150). In addition, carriers of a rare variant in CETP have greater CETP activity and decreased HDL levels along with increased sepsis mortality (152).

Several studies have also examined the amounts of LDL-C, total cholesterol (TC), triglycerides (TGs), and apoproteins during sepsis (144, 145, 153). LDL-C, TC, apoA-1, and apoB were significantly decreased in sepsis nonsurvivors and decreased apoA-1 predicted sepsis-related 30-day mortality (142). TC, VLDL, and apoA-1 concentrations are significantly increased from day 1 to day 7 in pediatric patients with sepsis (153). Moreover, TC is negatively correlated with C-reactive protein, a well-known inflammatory biomarker of sepsis (153). Another study demonstrated an association of decreased TG levels with sepsis nonsurvival (145). Furthermore, adding TG levels to SOFA score improves the predictive accuracy of 28-day mortality (145). However, the utility of plasma TG as a prognostic marker is controversial, as Sharma *et al.* (154) found that TG concentrations do not change between sepsis survivors, sepsis nonsurvivors, and healthy controls.

Both LDL and HDL are important scavengers of toxins and bioactive lipids that can be taken up by the liver to provide protection. LDL uptake by the liver is dependent on membrane surface LDL receptor, which is reduced in the presence of proprotein convertase subtilisin/kexin type 9 (PCSK9). Walley and coworkers have shown human PCSK9 loss of function subjects in septic shock have improved survival and decreased inflammatory cytokine production (155). Furthermore, in mouse sepsis models, PCSK9 inhibition improved survival and reduced inflammation, which was not observed in similar experiments performed with LDL receptor–deficient mice. HDL has also been tested as a sepsis therapeutic in animal models. Recombinant high density lipoprotein (rHDL) reconstituted with apoA-1 Milano administered to endotoxin-challenged rats attenuates hepatic and renal dysfunction and enhances the anti-inflammatory and antioxidant capacity (156). Tanaka *et al.* (157) demonstrated that HDL improved survival rates, reduced inflammation, and enhanced bacterial clearance in animal models. In addition, the apoA-1 mimetic peptide 4F improves survival rates and cardiac performance in septic rats (158, 159). rHDL administered to experimental endotoxemic humans shows remarkable HDL elevation in plasma (160). Although these studies showing HDL protective actions in sepsis are promising, to date there have been no human sepsis studies conducted with either rHDL or mimetic peptides. However, there is a related ongoing phase I/II trial designed to stabilize cholesterol levels in sepsis and septic shock patients using a lipid emulsion containing fish oil (161, 162).

## Chlorinated lipids

Neutrophils are early mediators of the host immune response during sepsis. The oxidants produced during neutrophil activation are important for bacterial killing, and they also have a role in organ damage through the release of reactive oxygen species (8, 163, 164, 165, 166). These reactive oxygen species target host tissue lipids. The chlorinated lipidome represents an important group of lipids produced by neutrophil-derived reactive oxygen species. Neutrophil-derived myeloperoxidase (MPO) produces hypochlorous acid (HOCl), a strong halogenating and oxidizing agent that reacts with both microbe and host molecules. The Ford group discovered that MPO-derived HOCl targets the vinyl ether bond at the *sn*-1 position of plasmalogen lipids (167). Plasmalogen vinyl ether oxidation by HOCl liberates 2-chlorofatty aldehyde (2-ClFALD) (168, 169), which is the precursor of other members of the chlorinated lipidome including 2-chlorofatty acid (2-ClFA) and 2-chlorofatty alcohol (170, 171).

Chlorinated lipids have been examined in both animal and human sepsis. Pike *et al.* (172) used a rat sepsis cecal slurry (CS) model that mimics early stages of human sepsis with antibiotic treatment and fluid resuscitation. Following CS treatment, rat sepsis nonsurvivors have higher plasma free 2-ClFA levels compared with survivors. Both free and esterified 2-ClFA are also increased in kidney, liver, lung, spleen, colon, and ileum of CS-treated rats. Moreover, exogenous 2-ClFA administration to rats results in loss of renal barrier function (172). Plasma 2-ClFA levels are also elevated in rat endotoxemia (173). In addition, the urinary metabolic clearance product of 2-ClFA, 2-chloroadipic acid, resulting from ω-oxidation and subsequent β-oxidation of 2-ClFA, is elevated in rats administered LPS (173). Brain and heart levels of 2-ClFALD have also been shown to be elevated in mouse endotoxemia (174, 175).

2-ClFA has also been investigated as a biomarker of human sepsis (176). Plasma levels of both free and esterified 2-chloropalmitic acid (2-ClPA) and 2-chlorostearic acid (2-ClSA) were assessed in the Molecular Epidemiology of Sepsis in the ICU cohort of human sepsis. Free and esterified forms of 2-ClSA and 2-ClPA plasma levels are significantly elevated in both sepsis survivors and sepsis nonsurvivors compared with healthy controls. Moreover, plasma 2-ClFA levels are elevated in patients who develop ARDS and 2-ClFA levels associate with 30-day mortality. When the plasma free 2-ClSA level is combined with the acute physiology and chronic health evaluation (APACHE) III score, ARDS prediction is significantly improved. Of interest, in septic neutropenic patients, plasma 2-ClFA levels are decreased, providing evidence that chlorinated lipid production is MPO dependent.

The potential roles of 2-ClFALD and 2-ClFA in sepsis and proinflammatory actions in the circulatory system have been investigated. Early studies demonstrated 2-ClFALD is a neutrophil chemoattractant and elicits endothelial P-selectin surface expression (168, 177). Subsequently, 2-ClFALD was shown to inhibit eNOS activity (178). 2-ClFA associates with endothelial Weibel-Palade bodies and mobilizes these granules, resulting in P-selectin surface expression, von Willebrand factor release, and angiopoietin 2 release. The mobilization of these granule contents results in increased neutrophil and platelet adherence, as well as loss of endothelial barrier function (179). 2-ClFA also induces neutrophil extracellular trap formation (180). Human monocytes treated with 2-ClFA undergo ER stress and produce reactive oxygen species (181). 2-ClFALD has also been shown to elicit brain endothelial barrier dysfunction (174) . However, the effects of 2-ClFALD on endothelial dysfunction and adhesion molecule surface expression is heterogeneous and varies with the tissue of origin of the endothelial vascular bed (182). 2-ClFA also elicits COX-2 expression in endothelial cells (183) and induces ER stress and mitochondrial dysfunction in brain endothelial cells (184). In vivo effects of 2-ClFA coupled with pharmacological manipulation have also been observed in the mesenteric microcirculation utilizing intravital microscopy (185, 186). Both 2-ClFALD and 2-ClFA elicit leukocyte rolling and adherence during superfusion in the mesenteric circulation (185). Furthermore, MPO inhibition with the pharmacologic inhibitor KYC reduces 2-ClFA levels, leukocyte rolling and adherence, and acute lung injury in CLP septic rats (186).

The role of chlorinated lipids in sepsis is a relatively new area of investigation compared with other lipids reviewed herein. 2-ClFA appears to be a good biomarker of sepsis outcomes that requires further multicenter evaluation and longitudinal studies. Furthermore, in addition to chlorinated lipids being biomarkers, they have profound effects at the level of the blood-vascular interface, which needs to be mechanistically explored as a therapeutic target to prevent microcirculatory collapse, organ failure, and mortality.

## Endocannabinoids

Although endocannabinoids have a long-established role in central nervous system function, significant roles have been identified in inflammation, cardiovascular function, and gastrointestinal function (187). Among nine endocannabinoid species identified so far, the two most extensively studied are anandamide (N-arachidonoylethanolamine: AEA) and 2-arachidonoylglycerol (2-AG) (188). AEA and 2-AG are found mainly in brain tissue; however, 2-AG can also be found in the gut (189, 190) . Both AEA and 2-AG bind to cannabinoid receptors CB1 and CB2, which are G protein–coupled receptors (191). CB1 receptors are enriched in the brain and spinal cord, whereas they are expressed at a lower degree in liver and pancreatic islet cells. CB2 receptors are found in immune cells such as macrophages and lymphocytes, with a reduced expression in neuronal cells (192).

The endocannabinoids have biological activity directed at the blood-vascular interface, which is critical in sepsis. 2-AG induces E-selectin and P-selectin on endothelial cells, resulting in leukocyte adhesion to endothelial cells, as well as promoting eosinophil chemotaxis and reducing endothelial cell viability (193, 194, 195). Endocannabinoids also have profound effects on immune cells (196); both 2-AG and AEA have immunosuppressive functions. AEA suppresses IL-12 and IL-6 production in monocytes (197), and endocannabinoids inhibit TNF-α release in LPS-treated microglia cells (198). Mestre *et al.* (199) demonstrated that AEA suppresses VCAM-1 and leukocyte migration through CB1 activation following viral infection in endothelial cells. Endocannabinoid metabolites also mediate inflammation, as AEA is hydrolyzed by fatty acid amide hydrolase into ethanolamine and AA, and the liberated AA serves as a substrate for COX-2, LOX, and P450, as discussed above (200). Fatty acid amide hydrolase mRNA levels in the whole blood of patients with sepsis is significantly reduced compared with healthy controls and remains low in sepsis mortality (201).

Endocannabinoid receptors have been examined as potential targets for sepsis therapeutics. Tschöp *et al.* (202) and Gui *et al.* (203) demonstrated that deletion of CB2 receptors results in increased mortality following CLP-induced sepsis or LPS-induced endotoxemia. Twenty-four hours following CLP sepsis induction, CB2-deficient mice have increased lung damage, neutrophil activation, and leukocyte recruitment in lungs (202). However, Kapellos *et al.* (204) did not observe a significant difference in neutrophil recruitment to the lungs following 2 and 8 h of LPS challenge in CB2 knockout mice. In these mice, splenic chemokines were increased following 2 h of LPS challenge, which was accompanied by transient neutrophil recruitment to the spleen (204). The conflicting data on neutrophil recruitment to the lungs with CB2 knockdown may be attributed to both the differences in the duration of the treatment and differences in bacterial sepsis and LPS endotoxemia (202, 204). On the other hand, pharmacological activation of CB2 receptors reduces sepsis-induced leukocyte adherence in the microcirculation, neutrophil recruitment to lungs, and lung damage in rodents (202, 205, 206). Deleting the CB2 receptor in LPS-treated or CLP-septic mice results in increased serum IL-6 and TNF-α levels, and CB2 agonism prevents the increases in proinflammatory cytokine levels in septic animals (202, 203, 206). Also, the CB2 agonist, HU308, decreases plasma levels of ICAM and VCAM in septic mice (206).

CB2 agonism may have a beneficial role in sepsis, whereas antagonism of the CB1 receptor seems to have a protective role (207). The CB1 antagonist, rimonabant, administered 4 h following the induction of CLP-sepsis in rats provides a significant increase in survival compared with vehicle-treated rats. CB1 blocking does not change sepsis-induced leukopenia, neutrophil migration, or plasma IL-6 levels. However, CB1 blockage in late-stage sepsis in CLP rats significantly increases plasma arginine vasopressin, a mediator associated with septic shock, multiple-organ failure, and death (208, 209). Thus, blocking CB1 may have a beneficial role in restoring patient blood pressure in late phase sepsis.

## Acylcarnitine and fatty acid metabolism

During sepsis, lipids are mobilized from adipose tissue and liver (210, 211). Accordingly, investigations have evaluated fatty acid intermediates as biomarkers of sepsis. Chung *et al.* (212) and Schmerler *et al.* (213) demonstrated short- and medium-chain acylcarnitines (C2–C10) are elevated in the plasma of patients with sepsis. The elevated plasma acylcarnitines are associated with SOFA score, hepatobiliary and renal dysfunction, thrombocytopenia, and hyperlactatemia (212). Plasma acetylcarnitine has a positive correlation with plasma IL-6, IL-8, and IL-10 as well as sepsis severity in patients (212). Plasma acylcarnitine levels may also provide a prognostic tool in human sepsis. Twenty-eight-day sepsis nonsurvivors have a 2-fold increase in plasma concentrations of cis-4-decenoylcarnitine (C10:1), hexanoylcarnitine (C6), butyroylcarnitine (C4) compared with sepsis survivors (214). Furthermore, increased plasma concentrations of medium-chain acylcarnitines differentiate sepsis from noninfectious SIRS (213). Elevated acylcarnitine levels detected during neonatal blood spot screening associate strongly with development of neonatal sepsis (215).

Mitochondrial function and fatty acid β-oxidation is compromised in human sepsis (216, 217, 218). In addition to elevated levels of acylcarnitines, elevated concentrations of tricarboxylic acid cycle metabolites such as lactic acid, pyruvate, and citric acid further confirm dysfunctional energy production due to mitochondrial defects (21, 214). Several investigators have studied the underlying molecular mechanisms of dysregulated lipid oxidation observed in sepsis (219, 220, 221, 222). Experimental animal sepsis identified decreased expression of fatty acid transport protein-2, fatty acyl-CoA synthase, carnitine palmitoyltransferase-1, medium-chain acyl-CoA dehydrogenase, acyl-CoA oxidase, and PPARs (219, 220, 221, 222). The role of PPAR-α is of particular interest, as leukocyte PPAR-α expression declines with the severity of sepsis in humans (223). In addition, in mouse CLP, liver PPAR-α is decreased, leading to increased plasma free fatty acids and hepatic lipotoxicity (211). The PPAR-α agonist pemafibrate reversed liver lipotoxicity following CLP sepsis (211). PPAR-α agonists also reduce LPS-elicited acute lung injury (223, 224). Septic PPAR-α-deficient mice also have reduced cardiac function and hepatic metabolic compensation compared with wild-type mice (225, 226). Although these studies suggest PPAR-α agonists to be potential human sepsis therapeutics, it is unclear whether altered PPAR-α levels are causal or coincidental (227). No clinical trials have been attempted with PPAR-α agonists in sepsis, which may be due to the cardiac, skeletal muscle, renal, and bone marrow toxicities caused by PPAR-α agonist in clinical trials for patients with atherogenic hypercholesterolemia or dyslipidemia (228).

## Concluding Remarks

Sepsis is the most common cause of death in hospitalized patients. The diverse sources of infection, the heterogeneity of immune and vascular responses, as well as multiorgan failure make sepsis extremely difficult to biochemically dissect to improve therapeutics. There is also a desperate need to improve early sepsis detection since mortality from sepsis increases 4% for every 1-h delay in sepsis diagnosis (229). Understanding the role of lipid biology during sepsis has the potential to provide new insights into the pathophysiology of sepsis and provide new therapeutic targets. This review has focused on lipid signaling molecules; fatty acid and cholesterol metabolism and transit via their macromolecular complexes; and new classes of lipids produced as a result of interactions with reactive oxygen species. As we move forward with future investigations focusing on the lipid biology of sepsis, it will be important to consider the limitations of animal models and consider studies that include human cells or organoids. There are considerable interspecies differences in genomes and innate and adaptive immunity responses, and within the human population itself, there is significant heterogeneity and variable comorbidities (230). Although animal models may not completely recapitulate human sepsis, they have been a key tool in understanding the pathogenicity of sepsis. Variability between different animal models (e.g., CLP, LPS administration, pneumonia models), the infection dosing strategy, and any clinical interventions such as antibiotics or fluid resuscitation may affect the identification of lipid-mediated pathology and lipid biomarkers. In addition, the diversity of sepsis sources, immune responses, and outcomes requires large sample sizes and multicenter studies in order to move forward with translational studies involving lipids. Finally, the integration of lipidomics with other omic platforms and big data sets from patient studies, in conjunction with artificial intelligence and computational modeling, should provide new insights in the lipid biology in sepsis.

## Conflict of interest

The authors declare that they have no conflicts of interest with the contents of this article.
